# Positive correlation between lipid accumulation product index and arterial stiffness in Chinese patients with type 2 diabetes

**DOI:** 10.3389/fendo.2023.1277162

**Published:** 2023-11-23

**Authors:** Jing Mao, Shenglian Gan, Quan Zhou, Fang Yu, Haifeng Zhou, Huilin Lu, Jing Jin, Qin Liu, Zhiming Deng

**Affiliations:** ^1^ Department of Medicine, University of South China, Hengyang, Hunan, China; ^2^ Department of Endocrinology, Changde Hospital, Xiangya School of Medicine, Central South University, Changde, China; ^3^ Department of Science and Education, Changde Hospital, Xiangya School of Medicine, Central South University, Changde, China

**Keywords:** lipid accumulation product, brachial-ankle pulse wave velocity, type 2 diabetes mellitus, arterial stiffness, insulin resistance

## Abstract

**Background:**

Many studies have confirmed that lipid accumulation products (LAP) predict arterial stiffness (AS) in hypertensive patients. But there is little research on the use of LAP in identifying early atherosclerosis in patients with type 2 diabetes mellitus (T2DM). The aim of this study was to determine the relationship between the LAP index and brachial-ankle pulse wave velocity (baPWV) in Chinese patients with T2DM.

**Methods:**

A total of 1471 Chinese participants with T2DM, ranging in age from 18 to 80, were included in this cross-sectional study. BaPWV measurements were used to calculate the AS. A baPWV greater than the 75th percentile (1700 cm/s) was defined as indicating increased AS. The LAP index was calculated from the combination of waist circumference (WC) and triglycerides (TG).

**Results:**

According to the quartiles of the LAP index, baPWV tended to increase after adjusting for sex and age. Multiple linear regression analysis showed that the beta coefficient (β) of baPWV increased by 31.0 cm/s for each unit of lnLAP that was increased, and the 95% confidence interval (CI) was (6.5, 55.5) cm/s. In multivariate logistic regression analyses, after fully adjusting for confounders, the risk of elevated baPWV increased with each unit increase in lnLAP, with an odds ratio (OR) of 1.3 (95% CI: 1.0, 1.8). According to the generalized additive model (GAM), we found that lnLAP was positively correlated with baPWV and baPWV elevation. The results were the same for males and females. Subgroup analyses showed that the positive correlation between lnLAP and elevated baPWV did not interact across all subgroups.

**Conclusions:**

In Chinese patients with T2DM, LAP was strongly and positively correlated with baPWV and elevated baPWV.

## 1 Introduction

Epidemiological surveys reveal that, globally, around 536.6 million persons aged 20 to 79 years had diabetes mellitus in 2021, with a prevalence of approximately 10.5%, and this figure is expected to climb to 783.2 million in 2045, with a prevalence of approximately 12.2% (1). This constantly rising incidence puts a significant strain on the economies of individual nations. Year after year, the challenging public health issue of diabetes prevalence grows. Type 2 diabetes mellitus (T2DM), which accounts for the bulk of the country’s diabetic population—more than 90% of all cases—is the most common type of diabetes in China, where the prevalence of the disease among those 18 and older has increased from 0.67% in 1980 to 11.2% in 2017 (2). A nationally representative cross-sectional survey with 173,642 participants in China in 2018 determined the overall prevalence of diabetes to be 12.4% (3).

Patients with T2DM are more likely than healthy people to develop insulin resistance (IR), hyperinsulinemia, lipid metabolism disorders, and elevated blood pressure, which can lead to vascular stiffness and associated cardiovascular disease (CVD) (4, 5). In addition, women with T2DM have faster atherosclerosis than men, especially after menopause (6, 7). Arterial stiffness (AS) is an early predictor of atherosclerosis and is generally considered a predictor of CVD incidence and mortality (8). Most current brachial-ankle pulse wave velocity (baPWV) measurements are now commonly used in research and clinical assessment of AS, and although they have a high degree of consistency, few time requirements, minimal operator dependence, and the ability to capture ankle-arm index waves simultaneously, they are still not universally available in rural areas, community hospitals, and large epidemiologic surveys in China. Because of the Chinese sedentary lifestyle and diet heavy in processed carbs and saturated fats, this can lead to disorders of lipid metabolism, obesity, and increased AS (9). Therefore, given that AS is a slow progression over a long period of time, we require a simple, cost-effective indicator to recognize early AS, especially for Chinese patients with T2DM.

Triglycerides (TG) are the standard lipid that is most strongly linked to AS, according to studies, and they are often linked to the early start of CVD (10). LAP is calculated from the combination of waist circumference (WC) and TG and differentiates between men and women by WC to better reflect central obesity and excessive lipid accumulation (11, 12). There are fewer studies on LAP and baPWV, and they have mostly been studied in the general population and hypertensive population in the past, and the results are controversial (13–15). As far as we know, there is little evidence that LAP can be used to detect early atherosclerosis in Chinese individuals with T2DM, so to fill this gap, we analyzed the correlation between LAP and baPWV in T2DM patients.

## 2 Methods

### 2.1 Characteristics of the population

All information was acquired between May 2020 and January 2022 at the Metabolic Management Center (MMC), Changde Hospital, Xiangya School of Medicine, Central South University, Hunan Province, China. which serves as a platform for standardized diagnosis and treatment of metabolic disorders and long-term follow-up (16). In this cross-sectional survey, 1665 diabetic patients aged 18 to 80 years were included. T2DM was identified using the 1999 World Health Organization diagnostic criteria: fasting blood glucose≥7.0 mmol/L, 2h postprandial plasma glucose≥11.1 mmol/L, or self-reported diabetes diagnosis (17). Exclusion criteria included age < 18 years (n = 3), patients with type 1 diabetes (n = 18), other types of diabetes (n = 4), pregnancy (n = 1), coronary artery disease (n = 52), stroke (n = 17), malignancy (n = 10), and patients with missing data for WC (n = 17), TG (n = 16), and baPWV (n = 56). After the exclusion of the above criteria, 1471 T2DM participants were finally included in this study.

### 2.2 Measurements of variables

All the anthropometric indices and socio-demographic parameters were measured and registered by trained researchers, and socio-demographic data were collected by questionnaire on gender, age, smoking, alcohol consumption, work status, salt intake (≤6 g/day, 6–8 g/day, and ≥8 g/day) (18), regular exercise (physical activity of moderate intensity at least three times per week) (11), duration of diabetes, history of CVD, and use of lipid-lowering, antihypertensive, or glucose-lowering drugs. Smoking (current, previous, and never) and alcohol consumption (current, previous, and never). Current smoking was defined as smoking more than one cigarette per day or more than seven cigarettes per week for more than six months. Former smoking was defined as meeting the above criteria six months ago and not smoking in the past six months. Current alcohol use is defined as drinking more than one “standard drink” (defined as 12 ounces of beer, 5 ounces of wine, or 1.5 ounces of distilled spirits) per week for more than six months. Former alcohol consumption is defined as meeting the above criteria six months ago and not drinking alcohol within the past six months (19). The anthropometric measurements include height, weight, WC, SBP (systolic blood pressure), and DBP (diastolic blood pressure). The participant’s height was measured with the shoes removed, and the three points of the head at the occiput, the ridge between the two shoulder blades, and the sacrum should be attached to the height measurement column. Weight (kg)/height^2^ (m^2^) was used to compute the body mass index (BMI). In order to calculate the WC, a non-elastic measuring tape was placed at the median point on a line from the superior margin of the iliac crest to the inferior margin of the costal arch (20). After sitting still for a minimum of five minutes, the subjects’ blood pressures were measured twice, and the final mean was calculated (21).

### 2.3 Laboratory assays

Laboratory data were obtained by drawing blood from each subject after at least 8 hours of fasting. We measured total cholesterol (TC), TG, high-density lipoprotein cholesterol (HDL-C), and low-density lipoprotein cholesterol (LDL-C). Fasting plasma glucose (FPG), fasting insulin (FINS), glycosylated hemoglobin (HbA1c), homeostasis model assessment for insulin resistance (HOMA-IR), and fasting C-peptide (FCP) were also measured. To evaluate post-load hyperglycemia, postprandial plasma glucose (PPG) was acquired from venous blood samples taken two hours after the steamed bread meal. The triglyceride glucose (TyG) index was calculated as the ln [TG (mg/dl) × FPG (mg/dl)/2]. The visceral adiposity index (VAI) was calculated as [WC(cm)/39.68 + (1.88 × BMI)] × (TG (mmol/L)/1.03) × (1.31/HDL-C (mmol/L)) for men and as [WC(cm)/36.58 + (1.89 × BMI)] × (TG (mmol/L)/0.81) × (1.52/HDL-C (mmol/L)) for women (15). The formula for calculating LAP varies by gender. For men, the formula used to determine the LAP was [WC (cm) - 65] × TG (mmol/L), and for women, the LAP was [WC (cm) - 58] × TG (mmol/L) (22). We corrected 66 cm for males with WC up to 65 cm and 59 cm for females with WC up to 58 cm in order to prevent LAP values that were not positive (14).

### 2.4 Assessment of baPWV

The technician performed baPWV measurements using an automated atherosclerosis detection device (model HBP-8000, Omron HealthCare (China) Co.). All subjects were required to rest for at least 5 minutes prior to the baPWV measurement, and the four cuffs of the automatic recording apparatus were wrapped around the elbow and ankle joints bilaterally. The band is fastened 2 cm above the inner ankle, while the arm band is fastened 3 cm above the elbow socket. The instrument uses the subject’s height as a reference and calculates the distance from the humerus to the ankle (La-Lb). The time difference of the waveform between the elbow and the ankle is denoted by Tba. The (La-Lb)/Tba formula was used to determine the baPWV (23, 24). In this study, we evaluated the mean of baPWV (25). Elevated baPWV, which was greater than 1700 cm/s in the study, was identified using the 75th percentile of baPWV measurement results (26, 27).

### 2.5 Statistical analysis

We ln-transformed LAP (lnLAP) because of the skewed distribution of LAP (14). To characterize the distribution of participants’ features, we divided them into four equal subgroups based on lnLAP levels. For continuous variables with skewed distributions, the median (1-3 quartiles) was utilized; for continuous variables with normal distributions, the mean ± SD was employed; and for categorical variables, numerical values (percentages) were utilized. ANOVA, or the Kruskal-Wallis H test, was utilized to assess differences in characteristics across lnLAP quartiles for continuous variables, while Fisher’s exact test, or the chi-square test, was employed to analyze categorical data.

We characterized the association between lnLAP and baPWV risk by β (beta coefficient) and 95% CI (confidence interval) of multivariate linear regression; meanwhile, multiple logistic regression demonstrated the correlation between lnLAP and elevated baPWV in T2DM patients by OR (odds ratio) and 95% CI. Five models were constructed by adjusting for covariates. Model 1 was not adjusted; Model 2 adjusts for gender and age based on Model 1; similarly, Model 3 added to Model 2 SBP, DBP, BMI, smoking, drinking, and work status. Model 4 added HbA1c, TC, HDL-C, and LDL-C to the previous model, and Model 5 added salt intake, regular exercise, glucose-lowering drugs, anti-hypertensives, lipid-lowering drugs, and duration of diabetes to the previous models. We compared the correlation between lnLAP and baPWV and elevated baPWV across genders by using the generalized additive model (GAM) dose-response relationship. Furthermore, we performed subgroup analyses to examine the relationship between lnLAP and higher baPWV by potential effect modifiers and did an interaction test. Statistical analyses for this study were performed using both R version 4.2.0 and EmpowerStats version 4.0.

## 3 Results

### 3.1 Participants’ characteristics

There were 1471 T2DM patients in the study, and the mean age of these participants was 51.81 ± 10.76 years, of whom 851 were men and 620 were women. **Table 1** displays the individual’s initial characteristics for lnLAP quartiles. Subjects with a higher lnLAP tended to have elevated BMI, WC, SBP, DBP, FPG, PPG, FCP, HbA1c, HOMA-IR, TC, TG, LDL-C, and baPWV. These subjects also had higher rates of smoking, alcohol consumption, participation in the workforce, use of lipid-lowering drugs, antihypertensive drugs, and high salt intake. Of the four groupings, subjects in the greater lnLAP category were younger and had lower HDL-C levels. In contrast, there were no statistically significant variations in the use of glucose-lowering medications or the length of diabetes between the four subgroups. The percentages of high baPWV in lnLAP quartiles were, respectively, 18.21%, 26.16%, 28.26%, and 26.09% in Q1, Q2, Q3, and Q4 (p<0.05). The proportion of men also grew, while the number of women declined as lnLAP increased.

**Table 1:** Baseline characteristics of the participants.

	lnLAP				
	Q1	Q2	Q3	Q4	P-value
N	368	367	368	368	
Age (years)	52.70 ± 9.90	53.17 ± 10.08	52.45 ± 11.32	48.91 ± 11.19	<0.001
Sex					0.029
Male	203 (55.16%)	199 (54.22%)	213 (57.88%)	236 (64.13%)	
Female	165 (44.84%)	168 (45.78%)	155 (42.12%)	132 (35.87%)	
BMI (kg/m^2^)	22.72 ± 2.32	25.16 ± 2.71	26.60 ± 2.93	28.09 ± 3.61	<0.001
WC (cm)	82.01 ± 7.55	90.36 ± 7.41	94.40 ± 8.03	98.14 ± 9.01	<0.001
SBP (mmHg)	131.05 ± 19.43	135.96 ± 18.10	136.87 ± 19.44	137.48 ± 19.66	<0.001
DBP (mmHg)	79.71 ± 10.92	83.27 ± 10.68	84.05 ± 10.75	86.28 ± 11.78	<0.001
FPG (mmol/l)	8.11 ± 3.13	8.58 ± 3.19	8.73 ± 3.31	9.84 ± 3.92	<0.001
PPG (mmol/l)	13.20 ± 5.69	13.41 ± 5.48	13.14 ± 4.86	14.54 ± 5.22	<0.001
Fasting C peptide	0.29 (0.20-0.39)	0.38 (0.25-0.50)	0.44 (0.28-0.63)	0.57 (0.40-0.76)	<0.001
HbA1c (%)	7.97 ± 2.37	8.35 ± 2.20	8.49 ± 2.16	8.81 ± 2.19	<0.001
HOMA-IR	2.27 (1.60-3.62)	3.44 (2.13-6.08)	4.29 (2.82-7.30)	5.67 (3.54-10.10)	<0.001
TC (mmol/l)	4.48 ± 0.98	4.82 ± 0.97	5.04 ± 1.14	5.47 ± 1.57	<0.001
TG (mmol/l)	1.07 ± 0.37	1.59 ± 0.42	2.32 ± 0.73	6.00 ± 6.28	<0.001
HDL-C (mmol/l)	1.42 ± 0.41	1.24 ± 0.29	1.19 ± 0.26	1.16 ± 0.26	<0.001
LDL-C (mmol/l)	2.46 ± 0.79	2.89 ± 0.83	3.02 ± 0.97	2.85 ± 0.90	<0.001
Duration of diabetes (month)	56.00 (15.00-109.00)	50.00 (14.00-98.00)	49.00 (17.00-108.00)	46.00 (14.00-94.75)	0.448
baPWV	1549.22 ± 310.62	1613.17 ± 332.63	1622.86 ± 319.50	1624.60 ± 316.14	<0.001
Smoking (%)					0.095
Never	252 (68.48%)	230 (62.67%)	230 (62.50%)	213 (58.04%)	
Former	24 (6.52%)	32 (8.72%)	32 (8.70%)	27 (7.36%)	
Current	92 (25.00%)	105 (28.61%)	106 (28.80%)	127 (34.60%)	
Alcohol consumption (%)					<0.001
Never	282 (76.63%)	260 (70.84%)	250 (67.93%)	212 (57.77%)	
Former	22 (5.98%)	23 (6.27%)	31 (8.42%)	28 (7.63%)	
Current	64 (17.39%)	84 (22.89%)	87 (23.64%)	127 (34.60%)	
Work (%)					<0.001
No	157 (42.66%)	163 (44.54%)	169 (46.05%)	116 (31.69%)	
Yes	211 (57.34%)	203 (55.46%)	198 (53.95%)	250 (68.31%)	
Salt intake					0.002
≤6 g/day	182 (49.73%)	150 (41.21%)	146 (40.11%)	136 (37.47%)	
6–8 g/day	168 (45.90%)	188 (51.65%)	182 (50.00%)	188 (51.79%)	
≥8 g/day	16 (4.37%)	26 (7.14%)	36 (9.89%)	39 (10.74%)	
Regular exercise					0.897
No	12 (3.26%)	10 (2.73%)	9 (2.45%)	9 (2.46%)	
Yes	356 (96.74%)	356 (97.27%)	358 (97.55%)	357 (97.54%)	
Glucose-lowering drugs					0.408
No	58 (15.76%)	63 (17.17%)	47 (12.77%)	55 (14.95%)	
Yes	310 (84.24%)	304 (82.83%)	321 (87.23%)	313 (85.05%)	
Antihypertensive drugs					0.006
No	294 (79.89%)	282 (76.84%)	258 (70.11%)	262 (71.39%)	
Yes	74 (20.11%)	85 (23.16%)	110 (29.89%)	105 (28.61%)	
Lipid-lowering drugs					<0.001
No	339 (92.12%)	338 (92.35%)	317 (86.38%)	296 (80.87%)	
Yes	29 (7.88%)	28 (7.65%)	50 (13.62%)	70 (19.13%)	
High baPWV					0.009
No	301 (81.79%)	271 (73.84%)	264 (71.74%)	272 (73.91%)	
Yes	67 (18.21%)	96 (26.16%)	104 (28.26%)	96 (26.09%)	
VAI	1.19 (0.24-4.14)	2.04 (0.78-6.18)	3.09 (1.55-8.29)	5.81 (1.95-51.18)	<0.001
TyG	1.34 (0.07-2.92)	1.82 (0.73-3.28)	2.18 (0.85-3.84)	2.96 (1.24-6.05)	<0.001

lnLAP, natural logarithm of lipid accumulation product; BMI, body mass index; WC, waist circumference; SBP, systolic blood pressure; DBP, diastolic blood pressure; FPG, fasting plasma glucose; PPG, postprandial plasma glucose; HbA1c, glycated hemoglobin; HOMA-IR, homeostasis model assessment for insulin resistance; TC, total cholesterol; TG, triglycerides; HDL-C, high-density lipoprotein-cholesterol; LDL-C, low-density lipoprotein-cholesterol; baPWV, brachial-ankle pulse wave velocity; VAI, visceral adiposity index; TyG, triglyceride glucose.

### 3.2 Correlation study of lnLAP and baPWV

**Figure 1:**
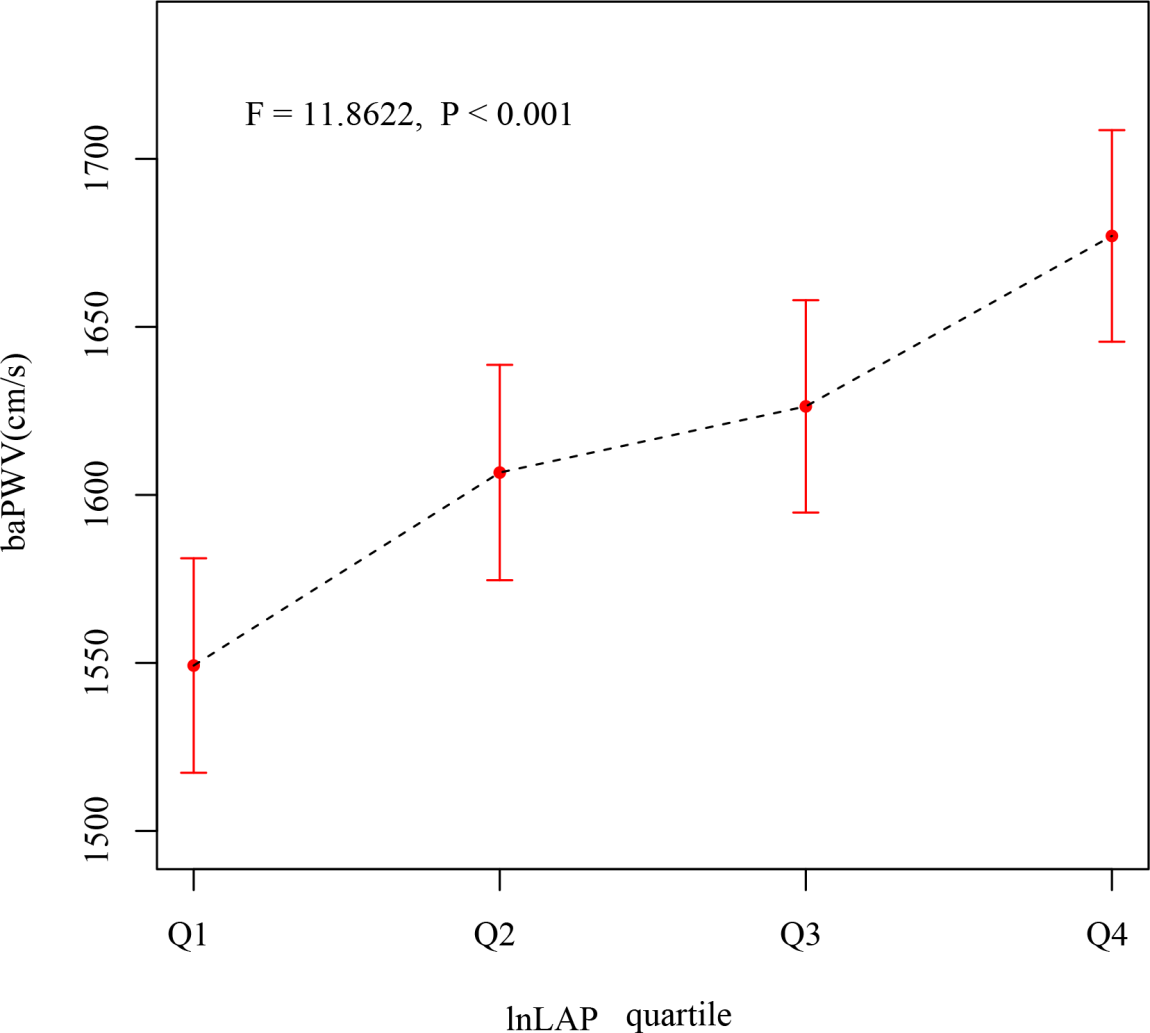
Comparison of baPWV after adjusting lnLAP quartiles for age and sex.

After correcting for sex and age, as seen in **Figure 1**, the mean baPWV of subjects showed an increasing trend in the lnLAP index quartile (F = 11.8622, P < 0.001). The mean baPWV values from the different lnLAP groups (quartiles 1–4) were 1550 (1517, 1582), 1605 (1573, 1638), 1629 (1597, 1661), and 1675 (1643, 1707) cm/s (P <0.001), respectively.

**Figure 2:**
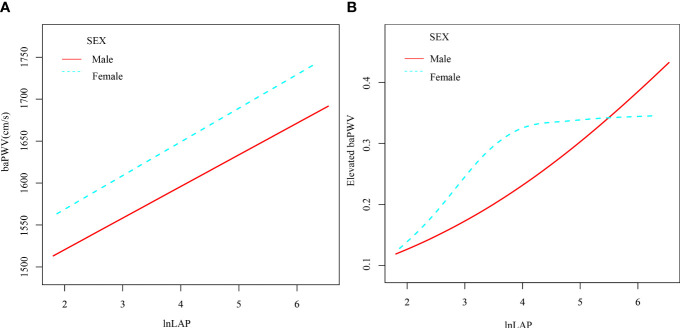
Generalized Additive Modeling of the relationship between lnLAP and baPWV **(A)** and elevated baPWV **(B)** across sexes after adjustment for age, SBP, DBP, BMI, smoking, alcohol, work, salt intake, regular exercise, HbA1c, TC, HDL-C, LDL-C, glucose-lowering drugs, anti-hypertensives, lipid-lowering drugs, and duration of diabetes.

A 1-unit increase in lnLAP in the logistic regression model was linked to a 31.1 (95% CI: 6.5, 55.9) cm/s increase in the β coefficient of baPWV after accounting for confounding variables (**Table 2**). We further grouped the lnLAP quartiles and placed them as categorical variables in a logistic regression model; in model 5, adjusting for all confounders, the β coefficient of the baPWV increased by 75.4 (95% CI: 22.6, 128.3) cm/s in the fourth group of the lnLAP compared with the first group, and in addition, we found that the baPWV’s β coefficient tended to increase between the lnLAP quartiles (P for trend = 0.007). It was also determined in the GAM that lnLAP was positively and linearly correlated with baPWV in both males and females (**Figure 2A**).

**Table 2:** Association between lnLAP and baPWV in different models.

lnLAP	baPWV, cm/s, β (95%CI)				
	Model I	Model II	Model III	Model IV	Model V
Per 1 unit increase	24.4 (6.0, 42.8)	49.5 (33.0, 66.0)	33.9 (15.7, 52.1)	36.3 (11.5, 61.0)	31.1 (6.5, 55.9)
Quartiles					
Q1	Ref	Ref	Ref	Ref	Ref
Q2	64.0 (17.7, 110.2)	57.4 (16.4, 98.5)	34.2 (-5.1, 73.6)	22.1 (-19.0, 63.3)	23.3 (-17.6, 64.1)
Q3	73.6 (27.4, 119.9)	77.1 (36.1, 118.2)	47.8 (6.2, 89.5)	34.9 (-10.3, 80.0)	28.2 (-16.8, 73.2)
Q4	75.4 (29.2, 121.6)	127.8 (86.5, 169.2)	89.4 (44.4, 134.4)	81.9 (29.0, 134.8)	75.4 (22.6, 128.3)
P for trend	0.002	<0.001	<0.001	0.003	0.007

Model I: adjust for None. Model II: adjust for age and sex. Model III: adjusts for age, sex, SBP, DBP, BMI, smoking, alcohol, and work. Model IV: adjust for age, sex, SBP, DBP, BMI, smoking, alcohol, work, HbA1c, TC, HDL-C, and LDL-C. Model V: adjusts for age, sex, SBP, DBP, BMI, smoking, alcohol, work, salt intake, regular exercise, HbA1c, TC, HDL-C, LDL-C, glucose-lowering drugs, anti-hypertensives, lipid-lowering drugs, and duration of diabetes.

lnLAP, natural logarithm of lipid accumulation product; baPWV, brachial-ankle pulse wave velocity; β, beta coefficient; CI, confidence interval.

### 3.3 Association between lnLAP and elevated baPWV

**Table 3** shows that for every unit rise in lnLAP in the fully adjusted regression model 5, the OR of elevated baPWV was 1.4 (95% CI: 1.0, 1.8). Using the lnLAP quartiles as categorical variables, the OR (95% CI) for elevated baPWV was 1.4 (0.9, 2.3), 1.5 (0.9, 2.5), and 1.9 (1.0, 3.4) for the quartiles 2, 3, and 4 of the lnLAP, respectively, compared with the quartile 1, after full adjustment. The increased risk of elevated baPWV was significant from the first to the fourth quartile (trend P = 0.046). Likewise, the GAM showed a significant positive correlation between lnLAP and elevated baPWV risk in both males and females (**Figure 2B**).

**Table 3:** Association between lnLAP and elevated baPWV in different models.

lnLAP	Elevated baPWV, OR (95%CI)				
	Model I	Model II	Model III	Model IV	Model V
Per 1 unit increase	1.2 (1.0, 1.3)	1.5 (1.3, 1.7)	1.4 (1.1, 1.7)	1.4 (1.1, 1.9)	1.4 (1.0, 1.8)
Quartiles					
Q1	Ref	Ref	Ref	Ref	Ref
Q2	1.6 (1.1, 2.3)	1.6 (1.1, 2.4)	1.5 (1.0, 2.3)	1.4 (0.9, 2.2)	1.4 (0.9, 2.3)
Q3	1.8 (1.2, 2.5)	1.9 (1.3, 2.7)	1.7 (1.1, 2.6)	1.6 (1.0, 2.6)	1.5 (0.9, 2.5)
Q4	1.6 (1.1, 2.3)	2.5 (1.7, 3.6)	2.1 (1.3, 3.3)	2.0 (1.1, 3.5)	1.9 (1.0, 3.4)
P for trend	0.011	<0.001	0.003	0.022	0.046

Model I: adjust for None. Model II: adjust for age and sex. Model III: adjusts for age, sex, SBP, DBP, BMI, smoking, alcohol, and work. Model IV: adjust for age, sex, SBP, DBP, BMI, smoking, alcohol, work, HbA1c, TC, HDL-C, and LDL-C. Model V: adjusts for age, sex, SBP, DBP, BMI, smoking, alcohol, work, salt intake, regular exercise, HbA1c, TC, HDL-C, LDL-C, glucose-lowering drugs, anti-hypertensives, lipid-lowering drugs, and duration of diabetes.

lnLAP, natural logarithm of lipid accumulation product; baPWV, brachial-ankle pulse wave velocity; OR, odd ratio; CI, confidence interval.

### 3.4 Subgroup analysis by potential effect modifiers

**Figure 3:**
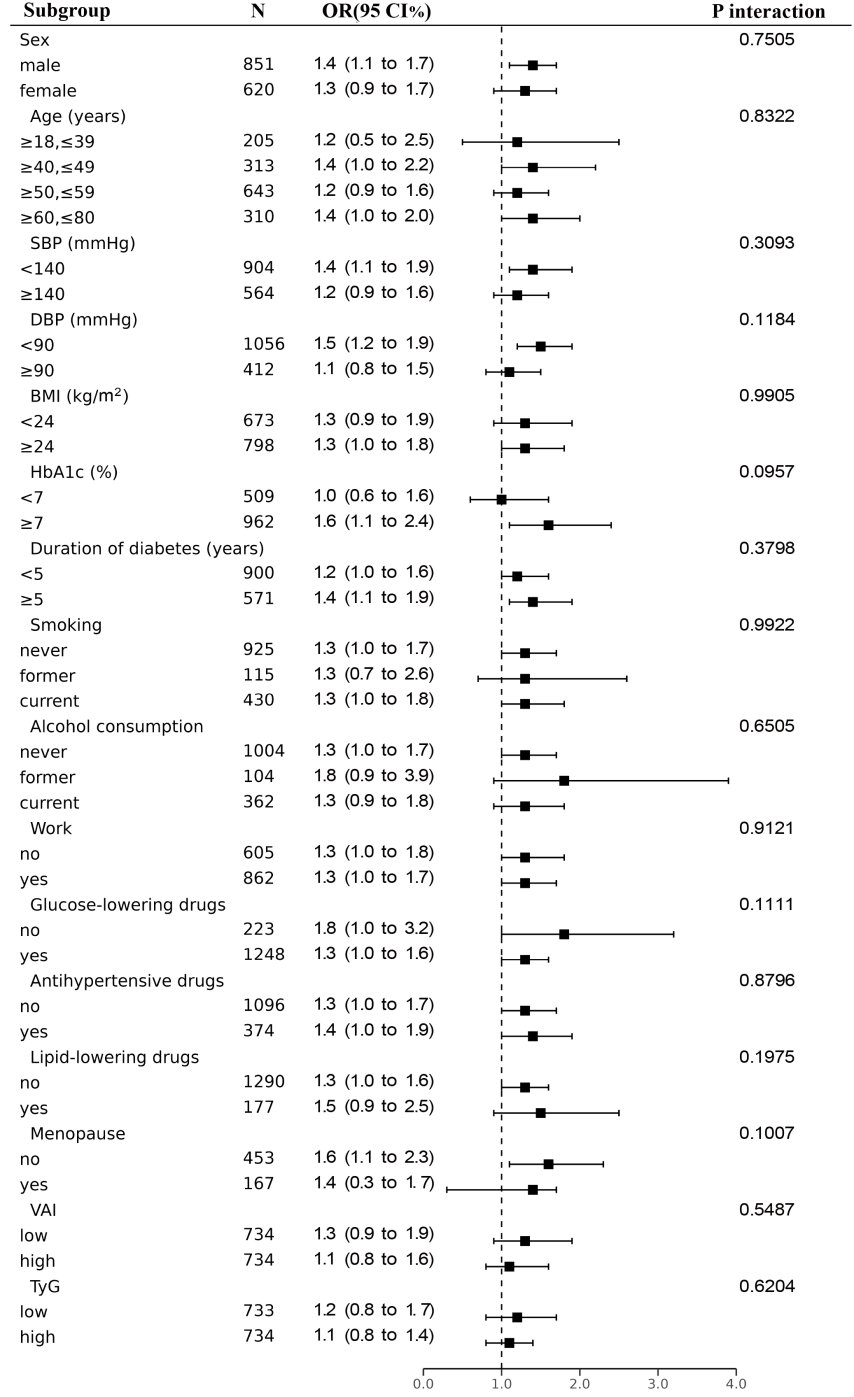
Subgroup analyses for the association between lnLAP and elevated baPWV were adjusted for age, sex, SBP, DBP, BMI, smoking, alcohol, work, salt intake, regular exercise, HbA1c, TC, HDL-C, LDL-C, glucose-lowering drugs, anti-hypertensives, lipid-lowering drugs, and duration of diabetes, except for the stratified variable.

To learn more about the connection between higher baPWV in each subgroup and lnLAP, we conducted a stratified analysis (**Figure 3**). The correlation between lnLAP and elevated baPWV was consistent in all subgroups. After excluding stratification variables and adjusting for remaining confounders, we found that lnLAP did not interact with baPWV in all subgroups. (P interaction > 0.05). Sex (P = 0.7505), Age (P = 0.8322), SBP (P = 0.3093), DBP (P = 0.1184), BMI (P = 0.9905), HbA1c (P = 0.0957), Duration of diabetes (P = 0.3798), smoking status (P = 0.9922), drinking status (P = 0.6505), work status (P = 0.9121), use of glucose-lowering drugs or not (P = 0.1111), use of antihypertensive drugs or not (P = 0.8796), use of lipid-lowering drugs or not (P = 0.1975), whether or not females were menopausal (P = 0.1007), low or high VAI (P = 0.5487), and low or high TyG (P = 0.6204).

## 4 Discussion

The present study demonstrated that LAP was positively linked with baPWV and elevated baPWV, and this positive association remained significant when adjusted for multiple regression, with a significant measured response between LAP and AS as assessed by baPWV. This is, as far as we are aware, the first assessment of an independent positive association between LAP and baPWV in Chinese T2DM patients.

There is a limited amount of pertinent research on the connection between LAP and AS, the majority of which has been done on members of the general population or hypertensive patients, and the conclusions are currently controversial (13–15). A study included 954 members of the general Japanese population aged 39 to 64 years and compared four lipid-related indices. It found no significant discriminatory power of LAP for increased AS (13). However, it was discovered that LAP was positively related to greater baPWV and was more significant in women in a different Japanese study of non-industrial workers aged 25 to 55 years (15).

Studies have shown that the mechanism by which LAP is associated with AS may be attributed to insulin resistance (IR). LAP is a stronger predictor of IR than obesity-related indices such as VAI and TyG (28). In addition, a Japanese study including 2818 healthy adults indicated that LAP was positively correlated with elevated baPWV and that LAP was superior to VAI and TyG (15). Our subgroup analysis with two equal groups of VAI and TyG was grouped as low and high. showed that LAP was positively correlated with elevated baPWV in both low and high VAI groups and TyG groups. Our study therefore reinforces the importance of LAP in predicting the risk of AS in patients with T2DM.

Furthermore, a study involving 4926 Chinese hypertensive patients, whose mean age was 64.42 years old, showed that LAP was positively associated with elevated baPWV and did not interact in gender subgroups (14), which is consistent with our findings. This study did not perform a subgroup analysis of menopausal and nonmenopausal women, and the population was mostly comprised of postmenopausal women. The predictive power of LAP in women could be diminished by low estrogen levels. It has been shown that men develop carotid atherosclerosis 10 years earlier than women, but the gap between the prevalence of carotid atherosclerosis in men and women narrows gradually after women’s menopause. This is associated with postmenopausal hormonal changes, oxidative stress, and changes in abdominal fat (29). Studies suggest that postmenopausal women may have dysregulated lipid metabolism, affecting body fat mass as well as altered abdominal fat distribution due to decreased estrogen levels and increased circulating androgen levels (30, 31).

The reasons for the inconsistent results of several of these studies may be related to variations in participant selection, racial differences, and the definition of AS. Given the controversial results of the above studies, additional studies and analyses of different populations are required to confirm the association between LAP and AS. Many meta-analyses have demonstrated that women with T2DM have faster atherosclerosis than men and are more susceptible to fatal coronary heart disease, myocardial infarction, and stroke, especially after menopause (6, 7, 32). Importantly, to our knowledge, most studies have only explored differences by gender and have rarely assessed differences between whether women are menopausal or not. Therefore, to add to this evidence, we performed an analysis in Chinese T2DM patients aged 18 to 80 years, and the findings revealed that LAP was positively linked with baPWV and did not interact in the gender subgroup after correcting for covariates. In addition, we further demonstrated by subgroup analysis that the positive correlation of LAP with baPWV had no interaction in the subgroup of whether women were menopausal or not. According to the Chinese definition of obesity, BMI <24 kg/m2 is considered non-obese and BMI ≥24 kg/m2 is considered overweight or obese (33), and we divided the subjects into two groups by BMI in the subgroup analysis. After adjusting for all confounders, it was shown that each one-unit increase in LAP increased the risk of AS by 1.3-fold in non-obese, overweight, and obese patients, suggesting that LAP was a better predictor of the risk of AS across all subgroups. Thus, even non-obese patients with T2DM should be closely monitored for LAP to reduce the risk of AS by lowering LAP or maintaining LAP at normal levels.

Although the mechanism by which LAP is associated with AS is unknown, many studies have suggested that it is likely to be attributable to IR (15), which predisposes the organism to a state of sub-clinical stress and induces a sustained chronic inflammatory response that leads to AS; IR increases the risk of AS after causing hyperinsulinemia (34–36). Physiological doses of insulin increase nitric oxide (NO) release via the phosphatidylinositol 3-kinase (PI3K)/Akt signaling pathway, which is blocked by IR (37). Elevated levels of IR and insulin activate vascular endothelial Na+ channels, leading to decreased NO utilization and atherosclerosis. Furthermore, IR activates the renin-angiotensin-aldosterone system. Elevated aldosterone and insulin both increase glucocorticoid kinase-1 (SGK-1) activity, which promotes hypertension, IR, and obesity, increasing the risk of CVD (35). Studies have also indicated that hyperinsulinemia with IR is also a risk factor for AS. In addition, the interaction of hyperglycemia and hyperinsulinemia exacerbates AS, which, depending on its pathophysiology, allows for the earlier development of hypertension and CVD in individuals with T2DM (38). LAP has been shown to have a high recognition of IR and is strongly associated with the development of T2DM, hypertension, and the metabolic syndrome (MetS) (28, 39, 40). Therefore, it is important to reduce LAP or maintain LAP at normal levels in individuals at risk for T2DM who are prone to a combination of multiple metabolic abnormalities.

However, our study included the following restrictions as well: First, because this study is cross-sectional, we are unable to draw conclusions about the causes of LAP and baPWV or rule out recall bias as a result of cross-sectional studies; future follow-up data from the MMC may provide more precise evidence. Second, this study was conducted only in Chinese T2DM patients, so the applicability of this study to other populations needs to be further verified.

## 5 Conclusions

To summarize, in patients with T2DM, our study found a significant positive correlation between LAP and baPWV. According to the findings, LAP can be utilized in epidemiological studies and actual clinical practice as a quick and accurate instrument for determining the risk of AS.

## Data availability statement

The raw data supporting the conclusions of this article will be made available by the authors, without undue reservation.

## Ethics statement

The studies involving humans were approved by the Medical Ethics Committee of the Changde Hospital, Xiangya School of Medicine, Central South University (Program number YX-2023-072-01). The studies were conducted in accordance with the local legislation and institutional requirements. Written informed consent for participation in this study was provided by the participants’ legal guardians/next of kin.

## Author contributions

JM: Formal Analysis, Methodology, Writing – original draft. SG: Project administration, Writing – original draft. QZ: Methodology, Writing – review & editing. FY: Data curation, Writing – review & editing. HZ: Data curation, Investigation, Writing – review & editing. HL: Investigation, Supervision, Writing – review & editing. JJ: Software, Supervision, Validation, Writing – review & editing. QL: Software, Supervision, Validation, Writing – review & editing. ZD: Conceptualization, Funding acquisition, Resources, Writing – review & editing.
